# Post-COVID-19 Thoracic Aortic Rupture with an Unforeseen Spinal Epidural Hematoma

**DOI:** 10.1055/s-0042-1757799

**Published:** 2023-02-27

**Authors:** Kush R. Lohani, Vikram V. Sannasi, Harvinder R. S. Sidhu, Oon C. Ooi, Wu P. Hung, Min Q. Chen

**Affiliations:** 1 Division of Vascular Surgery, Department of General Surgery, Ng Teng Fong General Hospital, National University Health System, Singapore, Singapore; 2 Department of Cardiac, Thoracic and Vascular Surgery, National University Heart Centre, Singapore, Singapore; 3 Department of Orthopedic Surgery, Ng Teng Fong General Hospital, National University Health System, Singapore, Singapore

**Keywords:** aortic rupture, aortitis, coronavirus disease 2019, spinal epidural hematoma

## Abstract

The importance of prompt diagnosis and early stenting of an aortic rupture cannot be overemphasized. We present a case of thoracic aortic rupture in a middle-aged gentleman who had recently suffered coronavirus disease 2019. The case was further complicated by the development of an unexpected spinal epidural hematoma.

## Introduction

Aortic rupture is usually attributed to a chronic generalized atherosclerotic aorta on a background of uncontrolled hypertension leading to aneurysmal dilatation or dissection. In the current coronavirus disease 2019 (COVID-19) pandemic, associations of COVID-19 with aortitis and aortic dissection have been reported. 1 2 Our case suggests a possible link between COVID-19 aortitis and aortic rupture. The patient's recovery was made more challenging by the rare complication of a spinal epidural hematoma via the extension of the mediastinal hematoma from the aortic rupture.

## Case Presentation

A 48-year-old man presented with acute urinary retention. Three months previously, he had suffered from left-side chest pain that resolved spontaneously. Aortic imaging was not performed at that time. He had also been found to have an asymptomatic COVID-19 infection. Moreover, he had had positive COVID-19 serology since 1 year back.

He was a nonsmoker and was recently diagnosed with hypertension. He had no limb weakness or chest complaints. His blood pressure was 169/112 mm Hg and heart rate 112 bpm. All peripheral pulses were palpable and symmetrical, and he had no peripheral neurological deficits. His symptoms were relieved with the insertion of an indwelling urinary catheter.

**Fig. 1:**
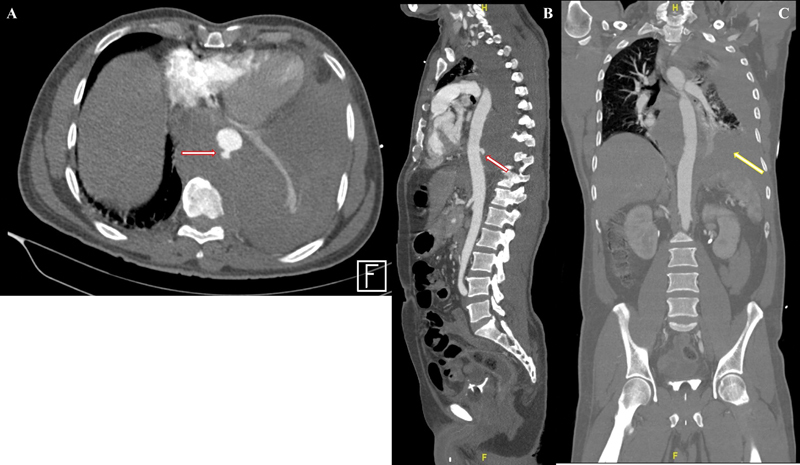
Computed tomography (CT) aortogram of the thoracic aorta **(A)** Axial section, **(B)** . Parasagittal section, **(C).** Coronal section). The red arrow shows the pseudoaneurysm of the descending thoracic aorta with the surrounding mediastinal hematoma and left hemothorax (yellow arrow).

Six hours later, he developed sudden onset left-sided chest pain with diaphoresis. Simultaneously, there was a significant drop in his blood pressure to 89/72 mm Hg with persistent tachycardia. Acute coronary syndrome was ruled out. Prompt computed tomography demonstrated a large left-sided hemothorax that was confluent with a large cuff of periaortic mediastinal hematoma. There was also a 0.9 × 0.8 cm outpouching noted in the posterior aspect of the descending aorta ( Figs. 1A–C ). Simultaneously, his serum hemoglobin dropped from an initial 12.5 to 9.9 g/dL.

**Fig. 2:**
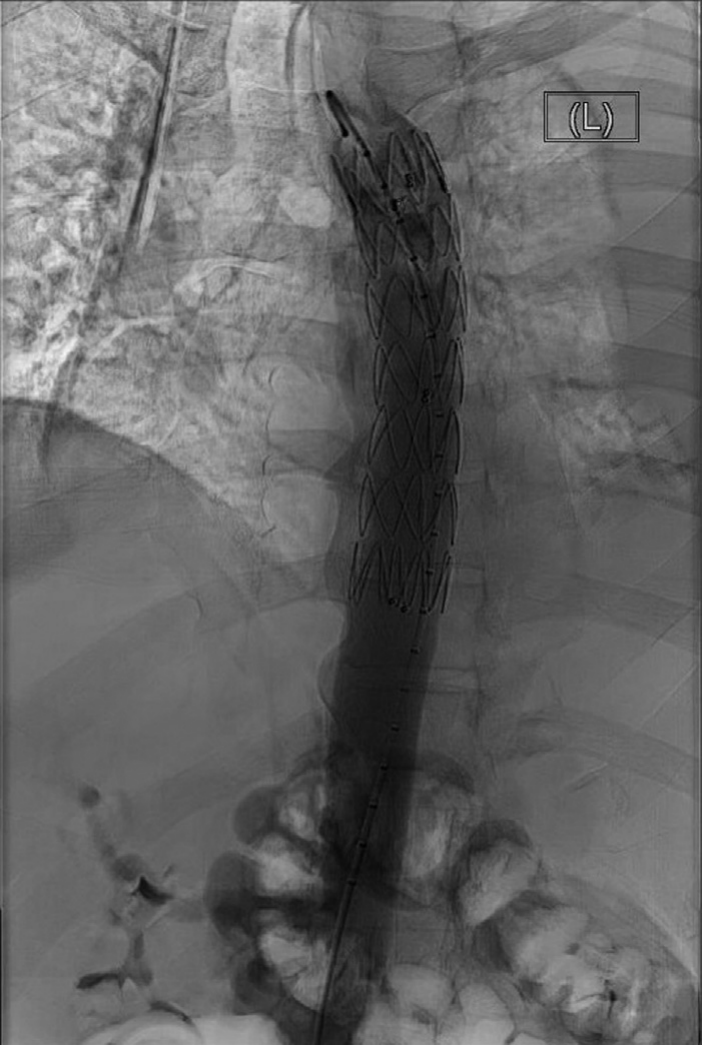
Completion angiography following the first thoracic endovascular aortic repair showing the well-stented descending aorta.

An emergent thoracic endovascular aortic repair (TEVAR) was performed using a 28 × 28 × 100 mm thoracic stent graft. A middescending thoracic aortic rupture was noted with no obvious true aneurysm or dissection. Completion angiography showed satisfactory stenting of the descending aortic ulcer with no evidence of endoleak ( Fig. 2 ). A left-sided tube thoracostomy drained 1.5 L of fresh blood.

Post-TEVAR and resuscitation, his blood pressure normalized; however, he remained persistently tachycardic with a gradual drop in his hemoglobin levels. His chest tube had stopped draining or oscillating and a chest X-ray demonstrated a persistent left hemothorax.

On day 2 of hospitalization, a video-assisted uniportal thoracoscopic surgical (VATS) procedure was performed. Approximately, 2 L of blood and 150 g of clot were evacuated. Generalized oozing was noted from the adventitia of the ruptured aorta. Hemostasis and irrigation of the pleural cavity were performed, and new chest tubes were placed. Post-VATS, however, persistent tachycardia and a gradual drop in hemoglobin levels were noted. There was a concern that the stent-graft may not be adequately covering the diseased descending thoracic aorta as there was ongoing adventitial ooze seen during the VATS, and it was not possible to determine “normal” versus “diseased” aortic wall on angiography.

**Fig. 3:**
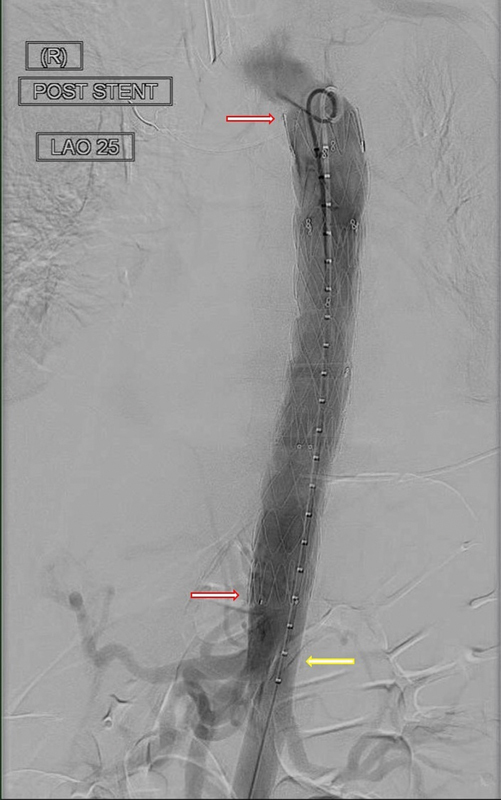
Completion angiography following the second thoracic endovascular aortic repair showing the extended stent graft (red arrows: proximal and distal extents) and the preserved celiac axis (yellow arrow).

On day 5, a repeat TEVAR was performed to extend the thoracic stent distally. Another 28 mm × 28 mm × 100 mm thoracic stent graft was deployed 3 cm proximal to the celiac axis, with 7 cm overlap of the original stent graft ( Fig. 3 ). After the second TEVAR, the patient's tachycardia eventually settled and his hemoglobin stabilized.

A second VATS was performed to evacuate the residual collection in the left hemithorax along with the placement of new large chest drains. The patient suffered interval development of a right-sided pleural effusion that was drained via an ultrasound-guided pig-tail catheter. He was treated with intravenous antibiotics and underwent intensive chest physiotherapy, incentive spirometry, and early ambulation. After 10 days, the patient was transferred out of ICU, with the removal of the chest drains. However, an initial trial of removal of a urinary catheter was not successful.

**Fig. 4:**
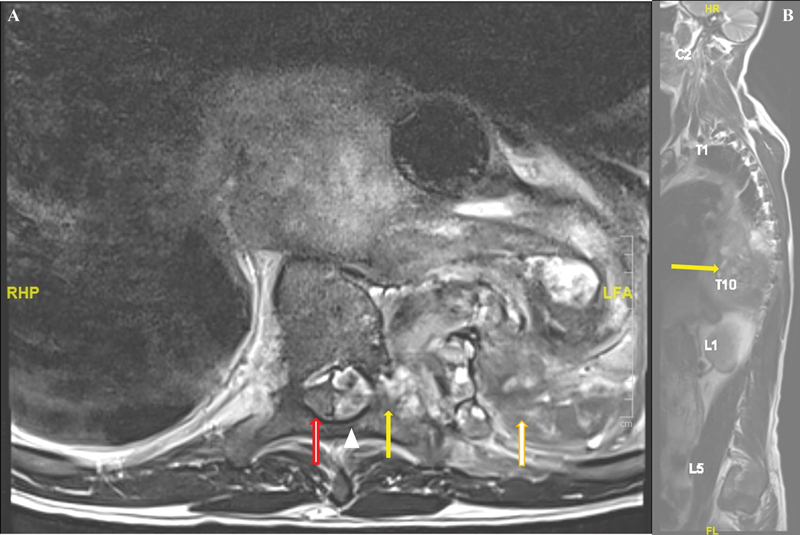
**(A)** Axial cut of the thoracic spine magnetic resonance imaging (MRI). Spinal cord (red arrow) was pushed to right by epidural hematoma (white arrowhead) which was derived from mediastinal hematoma (orange arrow) through the intervertebral foramen (yellow arrow). **(B)** Parasagittal cut of thoracic MRI whole spine. A large thoracic hematoma was seen encroaching into spinal elements (yellow arrow).

Three days after being in the general ward, the patient complained of weakness in his left lower limb. His left lower limb strength had decreased power (3/5) with intact anal tone and no saddle anesthesia. Urgent magnetic resonance imaging of the whole spine was done, revealing a hematoma in the left T7–11 epidural region that extended from the original large mediastinal hematoma via the left T8/9, T9/10, and T10/11 neural foramina ( Figs. 4A, B ). Urgent spinal cord decompression by microsurgical T7–11 laminectomy was performed.

Eventually, after multiple prompt and effective surgical interventions, as well as many sessions of physiotherapy, the patient flew back to his home with an intact aorta, good lung function, and restored power and function in both lower limbs, and without the urinary catheter.

## Discussion

Except from recently diagnosed hypertension, our patient had a thoracic aortic rupture without evidence of elevated serum cholesterol, atherosclerotic aorta, aneurysmal degeneration, or dissection. There was also no genetic predisposition for aortic pathology. Aortitis may have been a precursor to his aortic rupture given his persistently elevated erythrocyte sedimentation rate along with high initial white blood count and C-reactive protein. These suggest an acute on chronic inflammatory process. This inflammation could be due to either infective vasculitis, including COVID-19, 3 or some form of the rheumatological condition. However, no direct histological or microbiological investigation of the aortic wall was possible on this patient.

At present, no definite association of COVID-19 with aortic conditions has been established. 1 2 4 Our patient's recent COVID-19 infection raises the possibility of COVID-19 as the cause of his aortic rupture. Of note, all his septic work-up was negative, ruling out the possibility of a mycotic aneurysm.

The diagnosis of COVID-19-related aortitis should be one of exclusion. Aortic involvement in a noninfectious scenario is seen mainly in large vessel vasculitis such as giant cell arteritis 5 and Takayasu arteritis. 6 Our patient's presentation does not fit into this category, and his initial rheumatological work-up was also negative.

We believe that our case provides a possible link between COVID-19 aortitis and aortic rupture which has not been reported thus far. Endotheliitis and acute hypersensitivity vasculitis have been proposed to induce aortitis by COVID-19. 4 7 A patient with active COVID-19 had a focal infrarenal aortic dissection. 1 The patient displayed raised inflammatory markers along with negative rheumatological factors. The dissection resolved with timely steroid therapy. Similarly, steroid therapy has resolved aortic arch aortitis in a previously COVID-19-infected patient. 4

Our patient tested positive for COVID-19 3 months prior to this presentation. His background of COVID-19-positive antibody up to 1 year previously raises the likelihood that he was having repeated or persistent COVID-19 infections. His inflammatory markers were persistently raised, and his complaints of chest pain while he was COVID-19 positive could have indicated the onset of his aortitis. 1 It is possible that had our patient had earlier detection of any focal aortic pathology, the life-threatening thoracic aortic rupture could have been prevented.

Spontaneous spinal epidural hematoma due to extension of a mediastinal hematoma from a ruptured thoracic aorta has not, to our knowledge, been reported before. Spontaneous spinal epidural hematoma is associated with hypertension (21.4%), anticoagulation (22.8%), and antiplatelet therapy (10%). Anticoagulation has been implicated as the inciting factor. 8 Our patient lacked any obvious risk factors and there was no evidence of epidural vessel bleeding.

Our case consolidates the potential association of COVID-19 with aortic rupture and mandates a low threshold for consideration of aortitis in COVID-19-infected patients. The case findings are even more relevant at present, when COVID-19 is becoming endemic worldwide. The risks associated with long COVID-19 are as yet not fully known.
